# Frontal lobe hemodynamics detected by functional near-infrared spectroscopy during head-up tilt table tests in patients with electrical burns

**DOI:** 10.3389/fnhum.2022.986230

**Published:** 2022-09-08

**Authors:** Yoo Hwan Kim, Youngmin Kim, Jaechul Yoon, Yong Suk Cho, Dohern Kym, Jun Hur, Wook Chun, Byung-Jo Kim

**Affiliations:** ^1^Department of Neurology, Hallym University Sacred Heart Hospital, Hallym University College of Medicine, Anyang, South Korea; ^2^Department of Neurology, Graduate School, Korea University, Seoul, South Korea; ^3^Department of Surgery, Burn and Trauma Center, Daein Surgery and Medical Hospital, Seongnam, South Korea; ^4^Department of Surgery, Hangang Sacred Heart Hospital, Hallym University College of Medicine, Seoul, South Korea; ^5^Department of Neurology, Korea University Anam Hospital, Korea University College of Medicine, Seoul, South Korea; ^6^BK21FOUR R&E Center for Learning Health Systems, Korea University, Seoul, South Korea

**Keywords:** burns, electric, near-infrared spectroscopy, tilt table test, cerebral blood flow, hemodynamics

## Abstract

**Significance:**

Electrical burns can cause severe damage to the nervous system, resulting in autonomic dysfunction with reduced cerebral perfusion. However, few studies have investigated these consequences.

**Aim:**

To elucidate changes in prefrontal cerebral hemodynamics using functional near-infrared spectroscopy (fNIRS) during the head-up tilt table test (HUT) for patients with electrical burns.

**Approach:**

We recruited 17 patients with acute electrical burns within 1 week after their accidents and 10 healthy volunteers. The NIRS parameters acquired using an fNIRS device attached to the forehead were analyzed in five distinct HUT phases.

**Results:**

Based on their HUT response patterns, patients with electrical burns were classified into the group with abnormal HUT results (APG, *n* = 4) or normal HUT results (NPG, *n* = 13) and compared with the healthy control (HC, *n* = 10) participants. We found trends in hemodynamic changes during the HUT that distinguished HC, NPG, and APG. Reduced cerebral perfusion and decreased blood oxygenation during the HUT were found in both the NPG and APG groups. Patients with electrical burns had autonomic dysfunction compared to the HC participants.

**Conclusions:**

Using fNIRS, we observed that acute-stage electrical burn injuries could affect cerebral perfusion.

## Introduction

Electrical burns cause several unique clinical manifestations that differ from other burn injuries and require specific management. The assessment for clinical manifestations in patients with electrical burn is a challenging task, even for experienced burn specialists. The human nervous system has high electrical conductivity, and besides skin lesions, electrical injuries can cause severe damage to any part of the nervous system. Electrical injury has immediate and delayed effects in the nervous system. One of the immediate effects is autonomic nervous system (ANS) dysfunction and delayed effects can lead to myelopathy (Farrell and Starr, 1968; Ko et al., 2004; Nam et al., 2007). However, few studies have examined the functional changes in the brain, especially the ANS function, due to electrical injury (Brandon et al., 2009; Ramati et al., 2009). Burns are commonly known to have profound effects on an individual's psychological and emotional well-being (Bhatti et al., 2020). However, there is also a study reporting no significant difference in the incidence of post-burn anxiety or depression (Nilsson et al., 2019).

The head-up tilt table test (HUT) is widely used as a diagnostic tool to evaluate orthostatic intolerance (OI). However, monitoring blood pressure (BP) and heart rate (HR) during HUT is not sufficient to directly detect pathological dysfunction of the ANS (Szufladowicz et al., 2004; Wieling et al., 2004).

Recently, several optical techniques have been developed to help clinicians assess burn wound severity (Misgeld and Kerschensteiner, 2006; Kaiser et al., 2011; Thatcher et al., 2016) and measure neuronal activity in the brain (Kim et al., 2017). Electroencephalography (EEG), magnetoencephalography (MEG), and event-related brain potentials (ERP) provide good time resolution but limited spatial resolution. Positron emission tomography (PET), and functional magnetic resonance imaging (fMRI) have excellent spatial resolution but the limited temporal resolution (Irani et al., 2007). In comparison, functional near-infrared spectroscopy (fNIRS) technology is noninvasive, cost-effective, and can provide long-term real-time monitoring. fNIRS provides much better temporal resolution than fMRI. These advantages allow fNIRS to obtain better resolution of hemodynamic pathogenesis and allow for fast direct measurement of neural signals. The advantage of fNIRS over EEG/MEG/ERP is the spatial resolution that enables functional brain mapping as the number of emitters and detectors used in fNIRS (Jasdzewski et al., 2003; Franceschini and Boas, 2004).

fNIRS is an emerging technique that allows for monitoring of perfusion status in multiple areas. fNIRS technology has been used in research of brain-computer interface and functional neuroimaging in various neurological disorders, including Alzheimer's disease, schizophrenia, Parkinson's disease, functional connectivity, epilepsy, migraine, and stroke rehabilitation (Kim et al., 2017; Rahman et al., 2020). fNIRS can measure oxyhemoglobin (HbO) and deoxyhemoglobin (HbR) concentrations in the blood. Neural activity causes a physiological imbalance in the supply and use of oxygen, increasing the blood HbO concentration and decreasing the HbR concentration. Thus, fNIRS facilitates effective real-time measurement of cerebral perfusion by observing blood oxygen saturation changes. Our previous studies demonstrated the feasibility of direct cerebral perfusion monitoring with fNIRS during the HUT and Valsalva maneuver (Kim et al., 2018b, 2019).

This study was conducted to examine functional changes in the brain, detect autonomic dysfunction, and monitor prefrontal cerebral hemodynamics in patients with electrical burns using a commercially available fNIRS scanner during the HUT. Specifically, we wanted to compare the hemodynamic changes and psychological state after electrical burn injury between healthy controls and patients with electrical burns and between patients with electrical burns with and without autonomic symptoms. We hypothesized that fNIRS would demonstrate more precise and earlier changes in cerebral perfusion and blood oxygenation, and that depression and anxiety would be more commonly observed in patients with electrical burns.

## Methods

### Participants

Patients with electrical burns were recruited based on the following criteria: (1) electrical burn injury no more than 1 week before the examination; (2) no history of central nervous system disorders (stroke, Parkinson's disease, Alzheimer's, dementia); and (3) no history of significant head injury or alcohol/psychotropic drug abuse.

Demographic and clinical data, including age, sex, and comorbid chronic diseases, were also obtained. Additionally, healthy control (HC) volunteers who did not have any medical history that might have affected the ANS were recruited. Participants with severe arrhythmia were excluded because of the interference with HUT results.

All participants provided written informed consent before inclusion in the study. All procedures were performed in accordance with the Declaration of Helsinki and were approved by the Hallym University Institutional Review Board (IRB No. 2018-058).

### Study design

**Figure 1:**
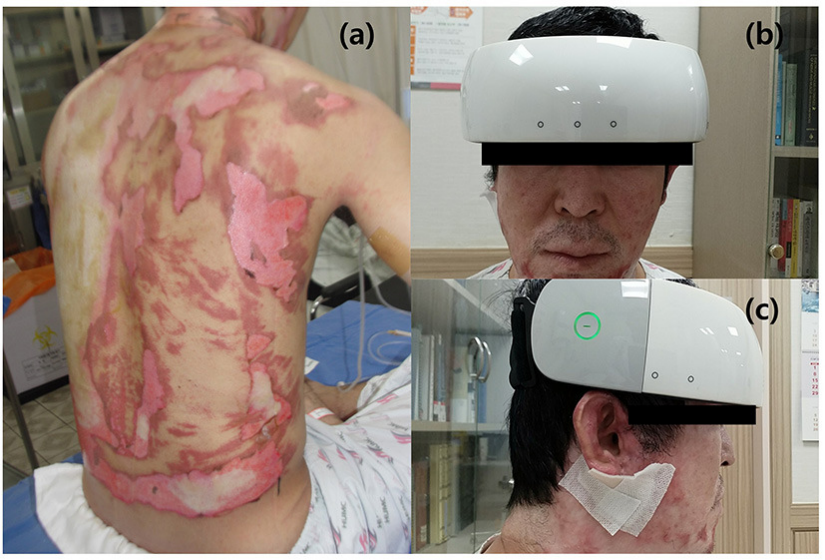
fNIRS device used in patients with electrical burns. **(a)** A patient with electrical burn injury. **(b,c)** Anterior **(b)** and lateral **(c)** views of a patient with electrical burn injury wearing the fNIRS device. **(c)** Cerebral hemodynamics were observed during the HUT using the fNIRS device attached to the frontotemporal area. fNIRS, functional near-infrared spectroscopy; HUT, head-up tilt table test. Patient informed consent was obtained for the use of personally identifiable photos for publication.

We conducted a prospective study from July 2018 to December 2020. All participants taking medications that affect autonomic function were asked to discontinue their medication for at least 24 h before the examination. HUTs were performed in a standard electrodiagnostic laboratory environment. First, the participants completed an autonomic dysfunction self-questionnaire, then the HUT was conducted after attaching the fNIRS device on the forehead to monitor cerebral hemodynamics (Figure 1).

### Self-rated questionnaires

Three self-rated questionnaires were used to assess the severity of autonomic dysfunction, anxiety, and depression. Autonomic dysfunction was measured using the Korean version of the Composite Autonomic Symptom Score 31 (K-COMPASS 31), consisting of 31 items that assess six different domains: four items for OI, three items for vasomotor, four items for secretomotor, 12 items for gastrointestinal, three items for bladder, and five items for pupillomotor functions. The minimum total score for the K-COMPASS 31 is 0, and the maximum score is 100. We scored only the four items related to OI separately, with the sum of these four items ranging from 0 to 10 (Sletten et al., 2012).

The Korean version of the Hospital Anxiety and Depression Scale (K-HADS) consists of seven items related to anxiety and seven items related to depression. A total subscale score of > 8 out of 21 possible points denotes considerable symptoms of anxiety or depression (Oh et al., 1999).

The Beck Depression Inventory (BDI) was developed to assess the type and degree of depression based on symptoms of depression. The questionnaire contains 21 questions about emotional, cognitive, motivational, physiological, and other symptoms. Possible scores range from 0 to 48, with higher scores reflecting more severe depressive symptomatology (Lim et al., 2011).

### HUT protocol

**Figure 2:**
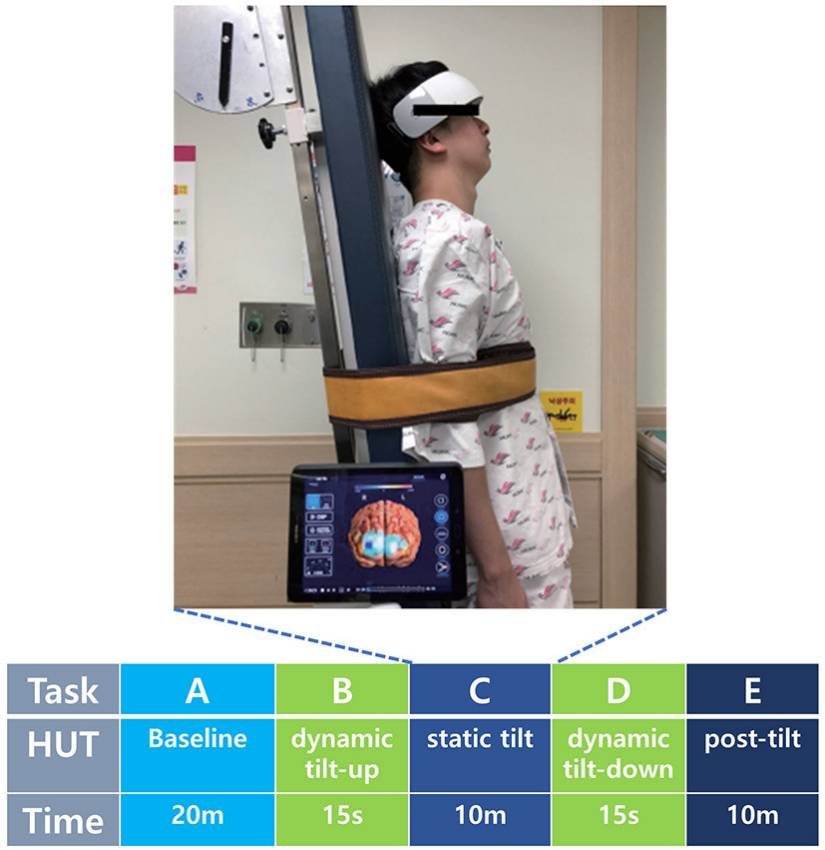
Cerebral hemodynamics were observed during the five HUT tasks using the fNIRS device attached to the frontotemporal area. HUT timeline consisted of baseline (Task A), dynamic tilt-up (Task B), static tilt (Task C), dynamic tilt-down (Task D), and post-tilt (Task E) phases. fNIRS, functional near-infrared spectroscopy; HUT, head-up tilt table test; m, minute; s, second. Patient informed consent was obtained for the use of personally identifiable photos for publication.

During the experiment, all participants underwent HUT with simultaneous fNIRS measurements. The HUT was performed using a sphygmomanometer cuff placed over the brachial artery for serial measurements of BP (systolic and diastolic) and HR. The fNIRS response to the HUT was divided into five phases: baseline, dynamic tilt-up phase, static tilt phase, dynamic tilt-down phase, and post-tilt phase. The HUT protocol was performed in a similar method with reference to previous studies (Sundkvist and Lilja, 1983; Jahan et al., 1996; Kim et al., 2020b). After a 20-min rest in the supine position, the baseline BP and HR were recorded (Task A). Then, each participant was slowly positioned at an angle of 70° on a standard, electrically driven tilt table with a footboard. Tilt the table from supine to 70° took 15 seconds at a typical tilt rate of 5°/s and vice versa (Task B and D) (Novak, 2011). In the static tilt phase, the participant was kept tilted for up to 10 mins (Task C) (Figure 2) (Low and Tomalia, 2015). The HUT was interrupted if presyncope-like symptoms occurred during the test. In the resting state of the post-tilt phase, the BP and HR were measured for 10 mins after the table had been returned to the supine position (Task E). Pharmacologic provocation tests were not performed.

### Patient groups

Based on the response pattern in the HUT, patients with electrical burns were grouped into the abnormal patient group (APG) (abnormal HUT results due to vasovagal syncope [VS], postural orthostatic tachycardia syndrome [POTS], or orthostatic hypotension [OH]) or the normal patient group (NPG) (normal HUT results despite electrical burns). We compared these two patient groups with each other and the HC group without electrical burns and normal HUT results.

APG diagnoses were defined as follows: Patients were diagnosed with OH if a reduction in systolic BP of at least 20 mmHg or diastolic BP of at least 10 mmHg was recorded within 3 min of standing up (Novak, 2016; Seok et al., 2018; Kim et al., 2020a). Patients were diagnosed with OHT if a postural increase in systolic BP by at least 20 mmHg was recorded (Freeman et al., 2011). Patients who displayed HR increases of more than 30 beats per min (bpm) or a maximum HR ≥ 120 bpm within the first 10 min without evidence of OH were diagnosed with POTS (Raj, 2013; Habek et al., 2019). Patients were diagnosed with VS if they exhibited spontaneous syncope associated with hypotension, bradycardia, or both (Freeman et al., 2011). As soon as syncope symptoms occurred, patients were quickly returned to the supine position.

We defined NPG as a group that had symptoms of OI not exceeding a 10% change in BP and HR relative to baseline, indicating normal HUT results.

Our screening procedure was strict. Participants who showed any deviation from the clinical definitions listed above were excluded from the study. This limited our pool of potential participants but provided the most qualified participants to analyze typical BP, HR, and fNIRS responses in each group.

### Devices and measurements

The fNIRS system consists of 24 laser diodes and 32 photodetectors, and 48 channels are measured with a 3cm-based source-detector pair. The fNIRS records data at two wavelengths, 780 and 850 nm, which are incident in the form of continuous waves with a sampling rate of 8.138 Hz. The light power is less than 1 mW and the total weight of fNIRS device is about 600g. The optical probes are connected to head with spring to minimize the pressure when the device is fastened to the forehead, and the rubber cap at the point of the optical probe minimizes signal contamination from the light noise of experiment environment. The gain of the detectors and the level of incident light power in each wavelength were calibrated before data measurement to ensure accurate signal acquisition while considering the difference of propagated light power caused by skull thickness, skin pigmentations, and other factors related light propagations. The measured data transmits wirelessly to the monitoring application through WIFI.

**Figure 3:**
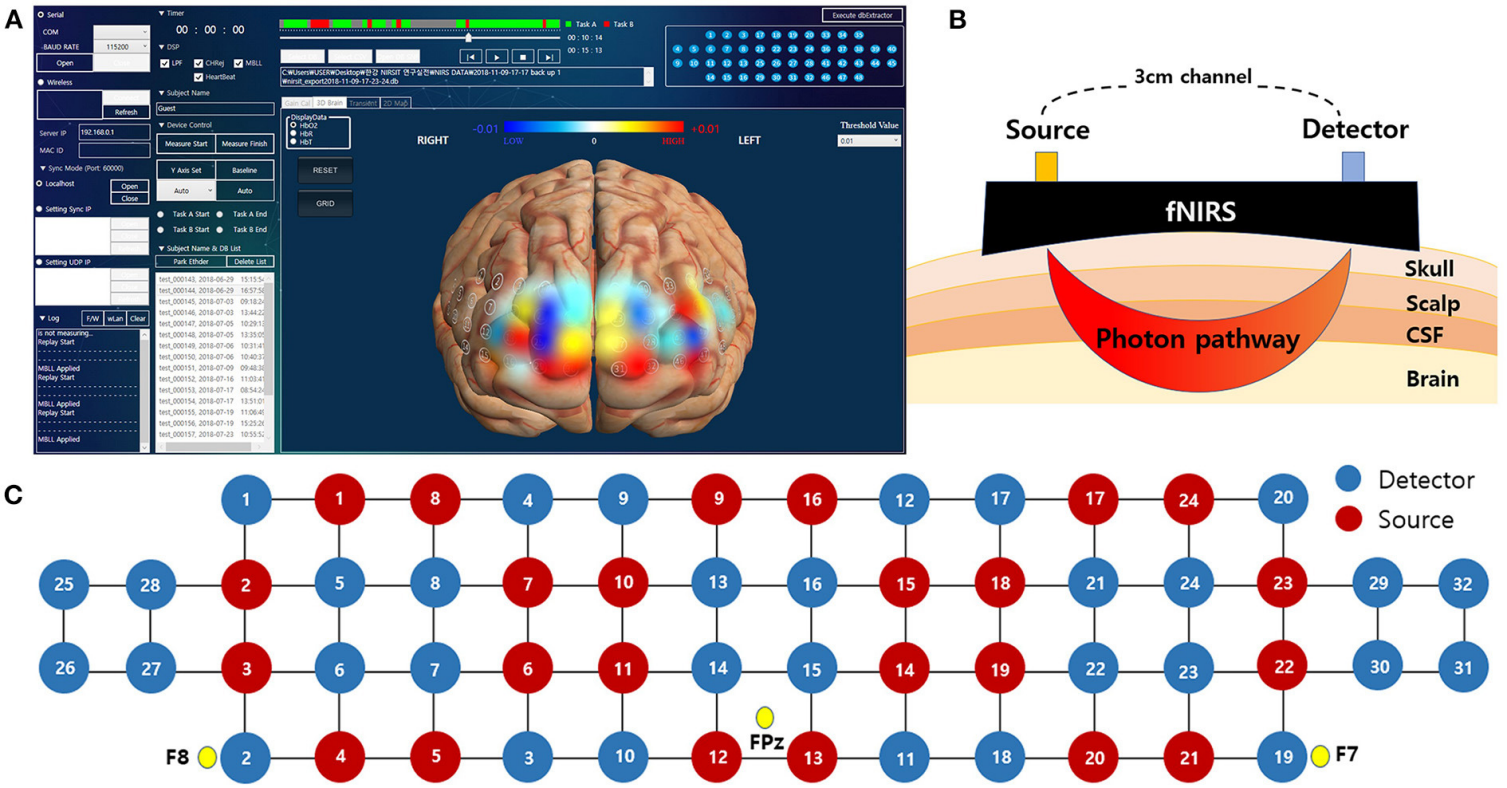
The fNIRS system used in this study. **(A)** An actual analysis program in which the measured data directly visualize the change in hemoglobin level as a color map on a 3-dimensional image. **(B)** Schematic diagram of fNIRS channels with different source-detector distance. **(C)** Spatial profiles of fNIRS channels with 10-20 system reference position. fNIRS, functional near-infrared spectroscopy.

Using the fNIRS system, hemodynamic signals in the prefrontal cortex were measured during the HUT. The FPz site of the international 10–20 system was used as a reference point for mounting the head device. We have attached the estimated MNI coordinates for each channel and Brodmann area in Supplementary material. fNIRS allows clinicians to monitor a patient's brain oxygenation in an intuitive way using 3D brain maps. The color shown in 3D brain images reflects the degree of oxygenation at each location in the brain at different time points during the HUT (Figure 3).

### Signal preprocessing and statistical analysis

The measured light signals were low-pass filtered with 0.1 Hz cutoff frequency to exclude the noisy channels contaminated by environmental light noise and physiological noise such as Mayer wave, respiration oscillation, and cardiac oscillation. To measure the change in blood flow according to the posture change, only the low-pass filter was applied without the high-pass filter. We used a discrete cosine transform based low-pass filter with a performance similar to a wavelet transform based filter. In addition, a 0.1 Hz filter was applied to measure the changes in posture, rather than rapid changes in blood flow such as pulse (about 1 Hz), respiration (0.2 Hz), and Mayer waves (about 0.1 Hz). The signal to noise ratio (SNR) calculated 10 to 15 sec of the baseline period in each wavelength and channels. If one of the channels had an SNR of less than 30 dB at a wavelength, that channel was rejected. The SNR formula is 20^*^log10 (mean intensity/deviation).

The optical density signal converted from the light signal can be distorted by motion artifacts that occur when the sensor slides because of head movement. To reduce motion artifacts, the tasks in our experiment were designed to prevent participants from moving their heads. Optical density signals were converted to changes in oxyhemoglobin concentrations (ΔHbO) and deoxyhemoglobin concentrations (ΔHbR) based on a modified Beer-Lambert law (Liu et al., 2000). A detailed review of fNIRS principles and terminology can be found in our previous article (Kim et al., 2019). Since the concentration change is a relative value, the average of the hemodynamic signals before initiation of each task was chosen as the baseline. We extracted block-averaged ΔHbO and ΔHbR signals from two trials for each participant. The regional representative value of HbO, HbR, and total hemoglobin concentrations (HbT = HbO + HbR) was extracted by averaging the categorized channels based on the specified region of interest.

We performed a statistical power analysis using G^*^Power 3.1 to obtain a suitable number of samples (Faul et al., 2009). Differences in demographic characteristics and questionnaire scores among the HC and patient groups were analyzed using either the Kruskal-Wallis test or Fisher's exact test. fNIRS parameters were calculated, and each patient group was compared to each other and to the HC group. After repeated measures of analysis of variance, each group's average HbO, HbR, and HbT values during the five HUT phases were analyzed using pairwise *post-hoc* comparisons. Statistical significance was set at *p* < 0.05, and data are presented as mean (range). Statistical analyses were performed using SPSS version 21.0 (IBM Corp., Armonk, NY, USA).

## Results

### Participant characteristics

**Figure 4:**
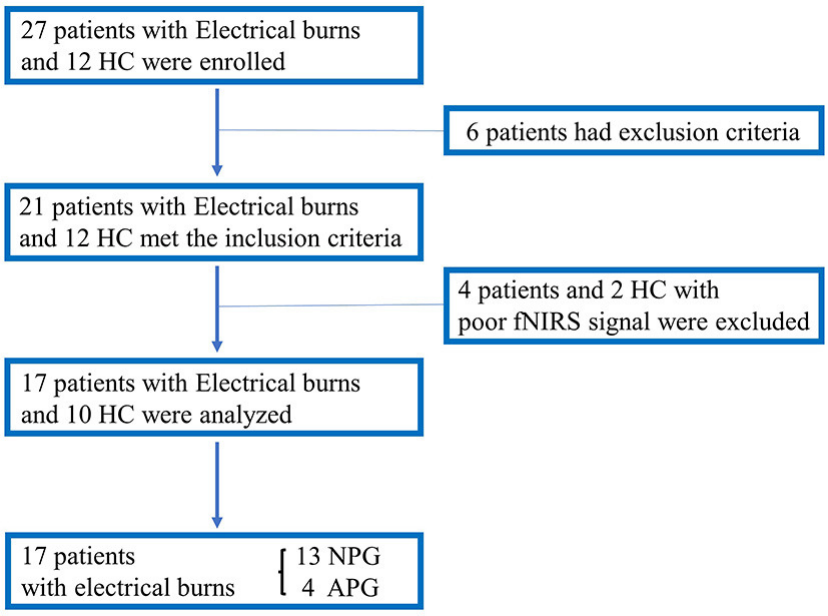
Flowchart showing enrollment of the study population. APG, electrical burn patients with abnormal HUT results; HC, healthy controls; HUT, head-up tilt table test; NIRS, near-infrared spectroscopy; NPG, electrical burn patients with normal HUT results.

Twenty-seven patients with electrical burns and 12 healthy participants were recruited for this study. However, six patients with electrical burns were excluded from the analysis because they had severe arrhythmia, which made BP/HR measurements unreliable. Four patients and two healthy participants were also excluded because of excessive motion artifacts in the fNIRS signal during the HUT. Finally, data from 17 patients with electrical burns and 10 healthy participants were analyzed (Figure 4).

Participants were grouped according to their characteristics and HUT findings as follows: (1) HC (*n* = 10; median age 37.3 years; 3 men); (2) NPG (*n* = 13; median age 44.8 years; 12 men); and (3) APG (*n* = 4; median age 50.8 years; 4 men). Thus, of the 17 patients with electrical burns, 13 had normal HUT results, whereas four patients had abnormal HUT results due to syncope in one patient, OH in two patients, and POTS in one patient. Tables 1, 2 present the demographic and clinical characteristics, including the HUT results of the three groups. Since many electrical burn accidents occur in industrial workers, the patients with burns enrolled in this study were predominantly men, and most of the injuries were high-voltage burns (Tables 1, 2).

**Table 1:** Demographic and clinical characteristics of the study population.

	**Study population (*n =* 27)**			
	**HC (*n =* 10)**	**NPG (*n =* 13)**	**APG (*n =* 4)**	***p*-value**
Age (y)	37.3 (26–75)	44.8 (23–60)	50.8 (32–68)	0.240
Male (*n*, %)	3 (30)	12 (92.3)	4 (100)	**0.002**
Height (m)	1.6 (1.5–1.8)	1.7 (1.6–1.9)	1.7 (1.7–1.8)	0.213
Weight (kg)	61.0 (42.0–85.0)	64.8 (45.6–91.5)	63.3 (53.8–72.0)	0.817
BMI (kg/m^2^)	22.2 (17.7–26.2)	22.7 (16.4–26.2)	21.3 (18.2–25.5)	0.741
K-COMPASS 31	2.0 (0–3)	12.0 (0–38)	5.0 (3–7)	**0.039**
Orthostatic intolerance	0 (0)	1.6 (0–5)	0 (0)	0.115
Beck depression inventory	3.3 (0–12)	14.0 (3–29)	7.5 (5–10)	**0.012**
HADS-anxiety	4.2 (0–10)	8.0 (3–12)	3.5 (3–4)	0.076
HADS-depression	3.5 (0–8)	9.8 (5–16)	7.0 (1–13)	**0.026**

APG, electrical burn patients with abnormal HUT results; BMI, body mass index; HADS, hospital anxiety and depression scale; HC, healthy controls; HUT, head-up tilt table test; K-COMPASS 31, the Korean version of the Composite Autonomic Symptom Score 31; NPG, electrical burn patients with normal HUT results. Data are presented as the mean (range) or number (%).

Statistically significant values (p < 0.05) are indicated in bold.

**Table 2:** Clinical characteristics of patients with electrical burns.

**Patient group**	**Case number**	**Sex**	**Age (y)**	**Height (m)**	**Weight (kg)**	**BMI (kg/m^2^)**	**TBSA (S2°/D2°/3°)**	**Voltage (V)**	**HUT result**
NPG	1	M	60	1.63	65.0	24.5	10 (3/4/3)	22900	Normal
	2	M	29	1.70	72.0	24.9	1 (0/1/0)	UK	Normal
	3	M	33	1.68	62.8	22.3	1 (0/0/1)	UK	Normal
	4	F	23	1.68	61.3	21.7	3 (UK)	320/220	Normal
	5	M	48	1.71	76.0	26.0	4 (UK)	6600	Normal
	6	M	49	1.68	68.0	24.1	1 (0/1/0)	22900	Normal
	7	M	52	1.74	55.0	18.2	3 (0/0/3)	UK	Normal
	8	M	55	1.67	54.0	19.4	1 (0/0/1)	20000	Normal
	9	M	56	1.62	58.1	22.1	10 (3/5/2)	22900	Normal
	10	M	50	1.66	71.0	25.8	15 (0/0/15)	22900	Normal
	11	M	47	1.63	62.0	23.3	2 (0/0/2)	22000	Normal
	12	M	55	1.67	45.6	16.4	15 (4/0/11)	22900	Normal
	13	M	26	1.87	91.5	26.2	6 (0/0/6)	UK	Normal
APG	1	M	32	1.83	65.0	19.4	6 (0/0/6)	UK	VS
	2	M	68	1.68	62.5	22.1	5 (0/2/3)	22900	OH
	3	M	57	1.72	53.8	18.2	6 (0/0/6)	22900	OH
	4	M	46	1.68	72.0	25.5	20 (15/5/0)	22900	POTS

APG, electrical burn patients with abnormal HUT results; BMI, body mass index; HUT, head-up tilt table test; NPG, electrical burn patients with normal HUT results; VS, vasovagal syncope; OH, orthostatic hypotension; POTS, postural orthostatic tachycardia syndrome; TBSA, total body surface area (%); UK, unknown.

The overall scores of the K-COMPASS 31 and the score of one of its subcategories, OI, were further analyzed. Although K-COMPASS 31 scores differed among the groups, there was no group difference in the OI domain (Table 1). There were also statistically significant score differences among the groups in the BDI and the depression domain of the HADS.

### fNIRS responses to the HUT

**Figure 5:**
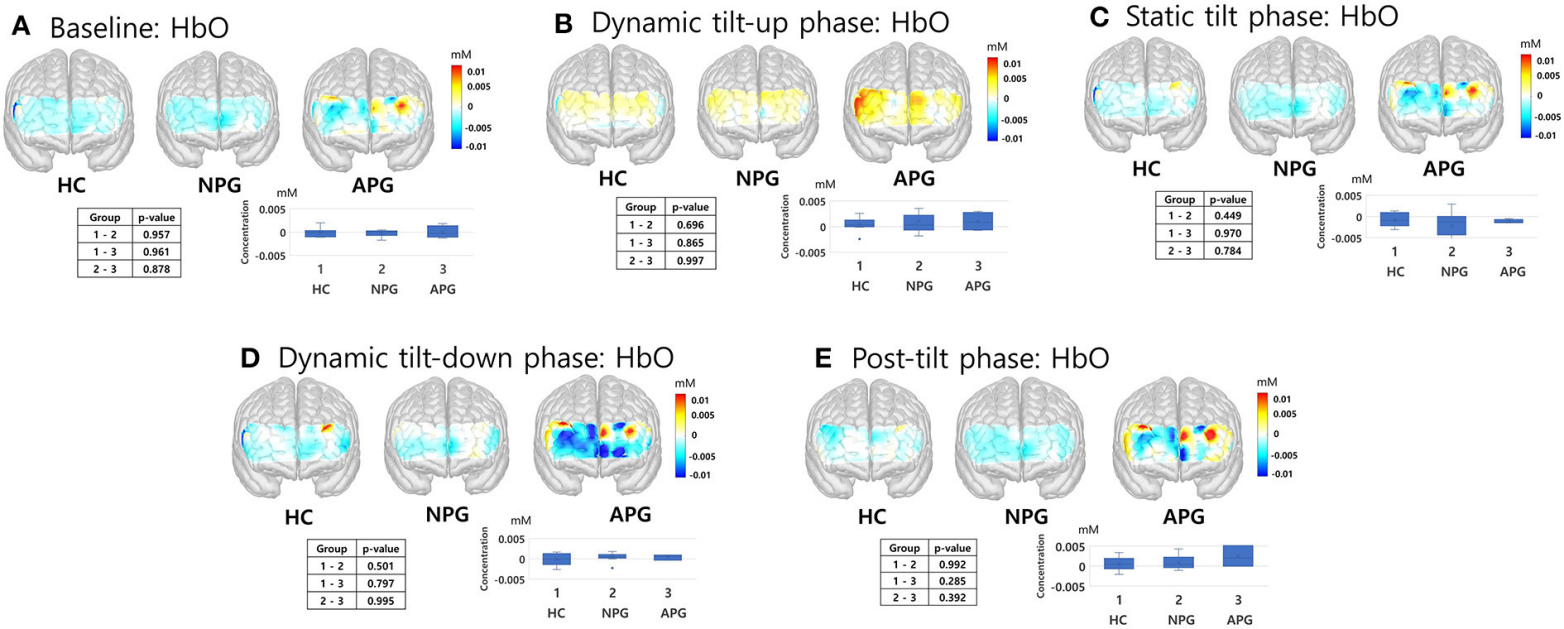
fNIRS responses of HC, NPG, and APG participants during the five HUT tasks. **(A)** Baseline, **(B)** dynamic tilt-up phase, **(C)** static tilt phase, **(D)** dynamic tilt-down phase, and **(E)** post-tilt phase. Kruskal-Wallis test was used for statistical analysis; statistical significance was set at *p* < 0.05. APG, electrical burn patients with abnormal HUT results; fNIRS, functional near-infrared spectroscopy; HbO, oxyhemoglobin; HC, healthy controls; HUT, head-up tilt table test; NPG, electrical burn patients with normal HUT results.

fNIRS responses to the HUT were divided into five phases: baseline, dynamic tilt-up phase, static tilt phase, dynamic tilt-down phase, and post-tilt phase. We analyzed the fNIRS data according to these five HUT phases and compared the hemodynamic responses (HbO, HbR, and HbT) during these phases. We aimed to determine whether fNIRS monitoring during the HUT phases added potential benefits to traditional BP/HR monitoring and investigated whether HC, NPG, and APG groups showed regional differences in blood volume changes over the prefrontal areas. The hemodynamic trends(HbO) among the groups during the HUT are depicted in Figure 5, which compares the average group hemodynamic response and the 3D maps from baseline to the post-tilt phase. We compared the 3D maps from baseline to post-tilt phase with the average group hemodynamic responses of HbR and HbT as well as HbO. However, we only present a 3D activation map of HbO as the hemodynamic responses (HbO, HbR, and HbT) were not statistically significant at any of the five HUT phases.

**Figure 6:**
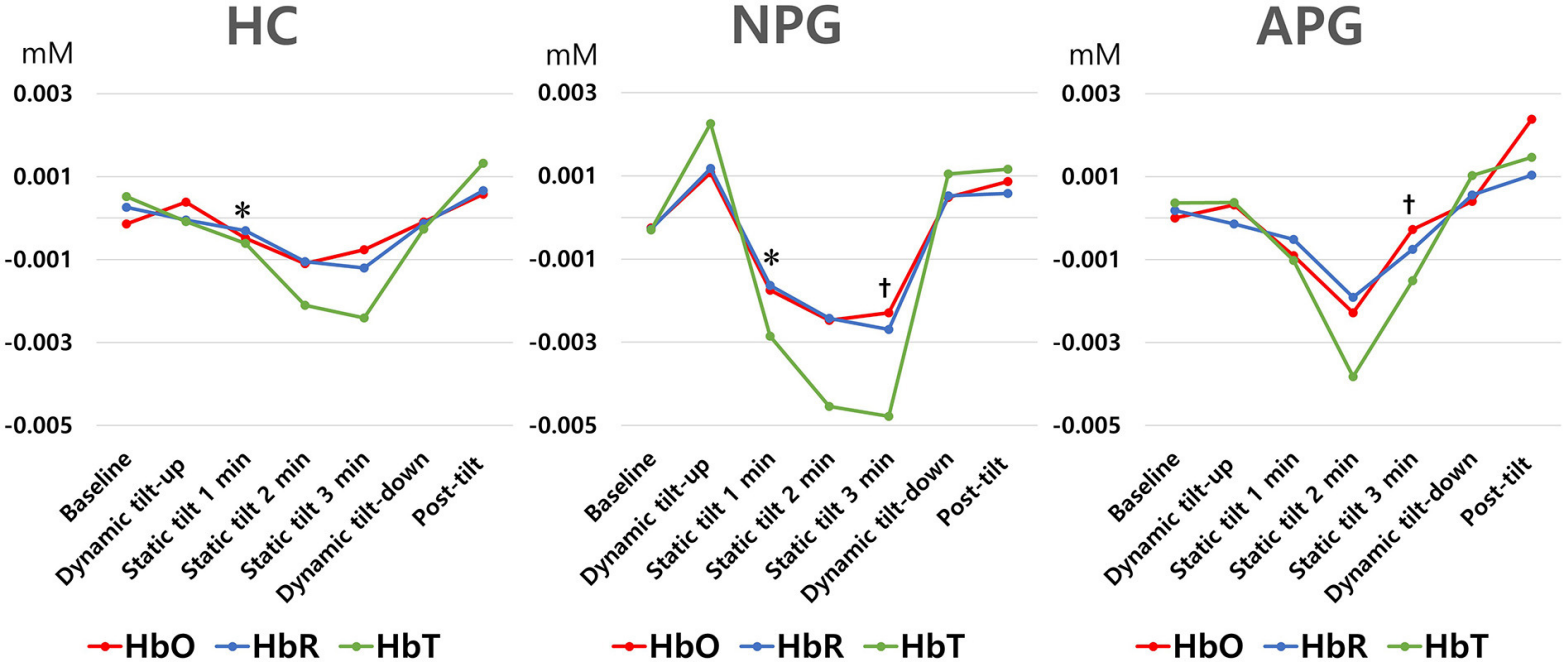
Differences in HbO, HbR, and HbT concentrations are shown for each group at 1-min intervals during the static tilt phase. Statistically significant differences (p-value < 0.05) among groups are denoted by footnote (*, HC versus NPG in HbO, , NPG versus APG in HbO). HbR and HbT concentrations were not different among groups. HbO, oxyhemoglobin; HbR, deoxyhemoglobin; HbT, total hemoglobin.

During the initial 3 min of the static tilt phase, the average changes in cerebral oxygenation were not significantly different among the groups. Further subdividing the static tilt phase into 1-min increments revealed significant differences in HbO concentrations in the 1-min and 3-min periods of the static tilt phase. *Post-hoc* analyses showed significant differences in HbO concentrations between HC and NPG at 1 min and between NPG and APG at 3 min of the static tilt phase (Figure 6; Table 3). HbT showed a similar tendency for the first 3 min of the static tilt phase without reaching statistical significance.

**Table 3:** Average HbO, HbR, and HbT values for each group during the five HUT phases.

	**HC**	**NPG**	**APG**	***p*-value**	***Post-hoc***
**HbO (μM)**					
Baseline	−0.142 (0.974)	−0.254 (0.714)	0.004 (1.550)	0.382	
Dynamic tilt-up	0.384 (1.310)	1.070 (2.380)	0.320 (1.670)	0.248	
Static tilt phase 1 min	−0.483 (1.380)	−1.750 (2.810)	−0.905 (0.818)	**0.024**	**a**
Static tilt phase 2 min	−1.100 (2.390)	−2.470 (4.650)	−2.280 (1.560)	0.110	
Static tilt phase 3 min	−0.762 (1.390)	−2.290 (3.490)	−0.271 (0.511)	**0.024**	**b**
Dynamic tilt-down	−0.097 (1.440)	0.484 (1.030)	0.410 (0.685)	0.263	
Post-tilt	0.575 (1.680)	0.866 (1.640)	2.400 (2.640)	0.651	
**HbR (μM)**					
Baseline	0.402 (0.798)	−0.028 (0.541)	0.181 (1.010)	0.312	
Dynamic tilt-up	−0.426 (1.660)	0.109 (0.578)	−0.465 (0.888)	0.136	
Static tilt phase 1 min	0.180 (1.110)	0.121 (0.710)	0.397 (2.290)	0.151	
Static tilt phase 2 min	0.051 (1.780)	0.049 (1.140)	0.369 (3.200)	0.151	
Static tilt phase 3 min	−0.439 (1.660)	−0.399 (0.950)	−0.482 (1.560)	0.729	
Dynamic tilt-down	−0.033 (1.360)	0.039 (0.239)	0.153 (0.993)	0.091	
Post-tilt	0.084 (1.500)	−0.286 (0.682)	−1.350 (2.160)	0.094	
**HbT (μM)**					
Baseline	0.259 (0.718)	−0.282 (0.622)	0.186 (0.500)	0.412	
Dynamic tilt-up	−0.041 (0.959)	1.180 (2.560)	0.517 (1.920)	0.222	
Static tilt phase 1 min	−0.303 (1.120)	−1.630 (2.530)	−0.508 (3.310)	0.064	
Static tilt phase 2 min	−1.050 (1.800)	−2.420 (4.520)	−1.910 (2.750)	0.064	
Static tilt phase 3 min	−1.200 (1.460)	−2.690 (3.430)	−0.753 (1.880)	0.052	
Dynamic tilt-down	−0.130 (1.270)	0.523 (1.090)	0.461 (0.776)	0.396	
Post-tilt	0.660 (1.940)	0.580 (1.570)	0.437 (0.681)	0.159	

Data are presented as means (standard deviation).

P-values were obtained using repeated-measures ANOVA.

Bold values denote statistical significance at P-value < 0.05.

The letters a-b indicate statistical significance for pairwise *post-hoc* comparisons after the repeated measures ANOVA: a: HC vs. NPG, b: NPG vs. APG.

APG, electrical burn patients with abnormal HUT results; HbO, oxyhemoglobin; HbR, deoxyhemoglobin; HbT, total hemoglobin; HC, healthy controls; HUT, head-up tilt table test; NPG, electrical burn patients with normal HUT results.

**Figure 7:**
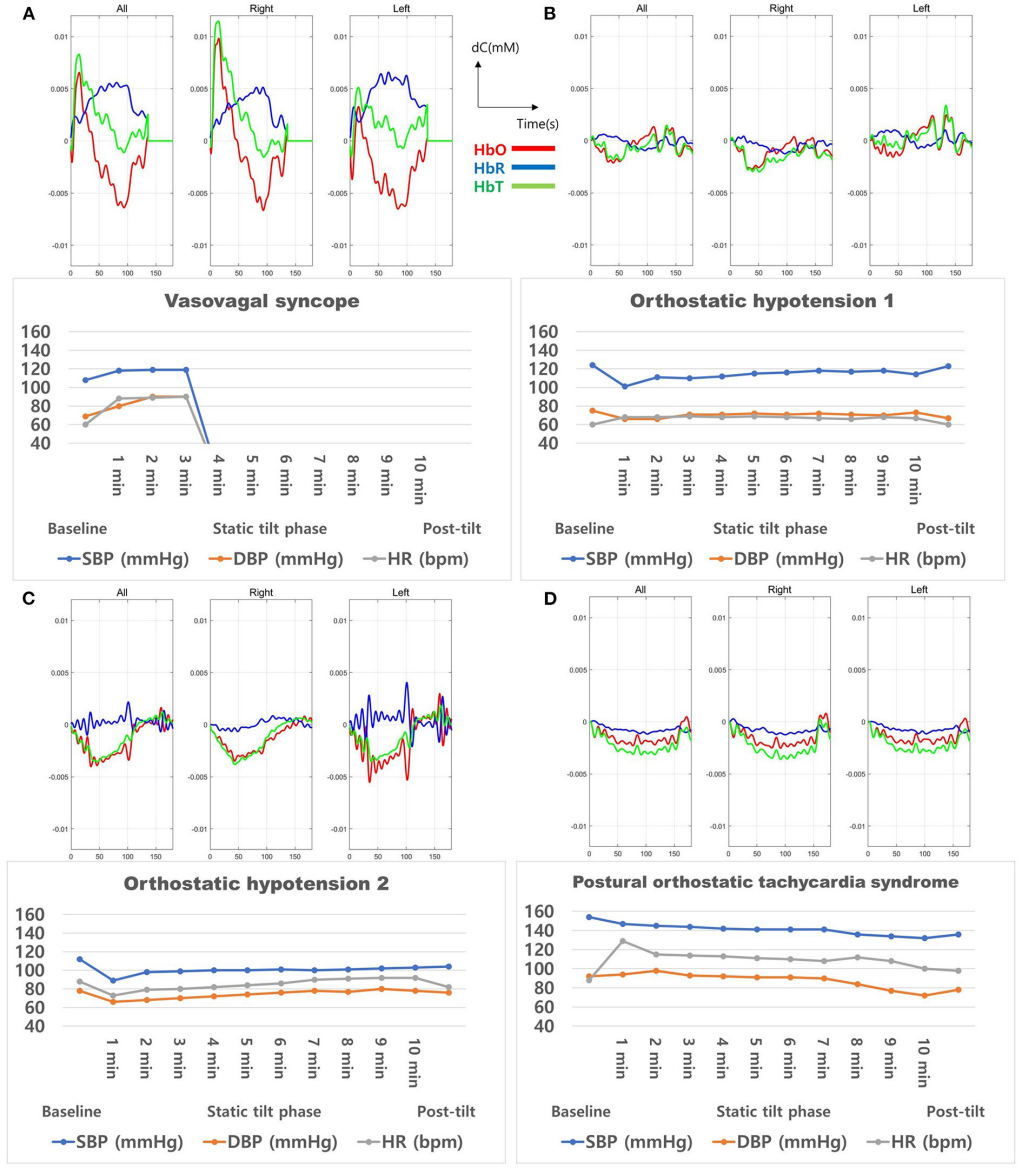
Cerebral oxygenation during the 3-min static tilt phase in burn patients with abnormal results in the head-up tilt table test. These four patients presented **(A)** vasovagal syncope, **(B)** orthostatic hypotension 1, **(C)** orthostatic hypotension 2, and **(D)** postural orthostatic tachycardia syndrome. HbO, oxyhemoglobin; HbR, deoxyhemoglobin; HbT, total Hb concentration.

Furthermore, each of the four APG cases was individually analyzed. The cerebral hemodynamics during the initial 3-min static tilt phase are presented in Figure 7, which shows the mean hemodynamic changes (HbO-red, HbR-blue, HbT-green) and HUT results of all/right/left channels for each APG case. The patient with syncope (Figure 7A) showed a significant drop in cerebral blood volume and blood oxygenation correlated with a decrease in BP and HR. Rapid BP and HR changes were formed between 2–3 min of the static tilt phase, whereas HbO and HbT changes occurred within 1 min of the static tilt phase. The two patients with OH (Figures 7B,C) exhibited initial drops in HbO and HbT concentrations, and one of these patients (Figure 7C) did not show any recovery toward baseline levels for HbO and HbT by the end of the static tilt phase. The patient with APG with POTS (Figure 7D) showed fluctuating Hb concentrations during the static tilt phase, which may have been due to a significant increase in HR influencing the fNIRS data. This patient also showed a gradual decrease in HbO and HbT concentrations in the static tilt phase.

## Discussion

Using fNIRS, we observed that electrical burn injuries affected cerebral perfusion and led to changes in blood oxygenation during the acute stage of electrical injuries. fNIRS data showed trends that differentiated HC, NPG, and APG based on hemodynamic changes during the HUT. In patients with electrical injury, reduced cerebral perfusion and decreased blood oxygenation were detected in both the NPG and APG groups. Furthermore, in contrast to HC participants, patients with electrical burns had clinical symptoms related to autonomic dysfunction and depression.

A diagnosis of OI has traditionally relied on BP and HR measurements by the HUT test. The NPG participants showed normal HUT results despite their electrical injuries; however, these results alone do not represent patients with burns without autonomic dysfunction or decreased cerebral perfusion since not all patients with burns have autonomic symptoms. Also, not all patients with OI have abnormal findings in the HUT. Our study findings suggest that the additional use of fNIRS may increase the diagnostic value of the HUT.

Previous studies have mainly focused on the first 3 min of the static tilt phase during HUT (Phillips et al., 2020). We designed this study with the expectation that the HUT would also exhibit characteristic cerebral hemodynamics in the dynamic tilt-up phase, dynamic tilt-down phase, and post-tilt phase. The mean value of cerebral hemodynamics for each task did not significantly differ among the study groups for any of the five HUT phases. In this study, patients with a relatively mild degree of burns, which could be tested for the acute stage HUT, were targeted, and the small APG group may be a factor that reduced the statistical significance of the hemodynamic difference among the three groups. However, analysis of the static tilt phase in 1-min increments revealed significant differences in HbO concentrations between HC and NPG and between NPG and APG groups. We previously demonstrated the potential of fNIRS to discriminate OI by providing characteristic signaling patterns during HUT (Kim et al., 2019). We found that BP and HR varied continuously at the beginning and end of the static tilt phase, and the concentrations of HbO, HbR, and HbT varied accordingly. It would be better if the time was further subdivided; however, for the convenience of analysis, the analysis was performed at 1-minute intervals. If analyses for each phase were performed at short time increments without averaging, there was a possibility that differences among the groups could have been revealed in other phases as well. Although only HbO showed statistical significance between groups, HbO representing blood oxygenation and HbT proportional to blood volume showed similar trends during the first 3 min of the static tilt phase. This was also observed in our previous study (Kim et al., 2018b, 2019). All three groups showed HbO increase in the dynamic tilt-up phase, presumably due to the pre-compensation mechanism for HbO decrease in the static tilt phase.

fNIRS demonstrated the potential to detect OI in APG before symptoms occurred. HbO and HbT fell in a pattern similar to the changes in BP and HR but showed a significant drop before HR and BP decreased, attributed to syncope. These findings are in agreement with the results of our previous studies on cerebral perfusion during syncope showing a gradual presyncope decrease in HbO and HbT (Kim et al., 2019). In addition to syncope, the OH and POTS cases also indicated a gradual decline in HbO and HbT concentrations in the static tilt phase. Notably, in two OH cases, HbO and HbT concentrations in one patient recovered above baseline during the second half of the static tilt phase; in contrast, there was no observable recovery above baseline until the end of that phase in the other patient. One case of POTS showed changes in HbO and HbT concentrations even without evidence of BP changes. These results demonstrated that patients with APG exhibited cerebral hemodynamic changes during the entire static tilt phase of the HUT and that fNIRS provided a more accurate status of cerebral blood flow than the observed BP and HR data. The current study findings suggest that fNIRS can be used for the early diagnosis of autonomic dysfunction in patients with electrical burns.

Systemic BP and cerebral blood flow are influenced by gravity. While standing, blood accumulates in the lower body parts. This leads to decreased venous return and cardiac output, which reduces cerebral blood flow and ultimately increases the oxygen demand of the brain (Robertson, 2008). When BP changes occur, baroreceptor reflexes regulate HR and vascular resistance through the sympathetic system and vagal tone. This neural activation causes a physiological imbalance between oxygen supply and demand, which increases the HbO concentration in the blood and decreases the HbR concentration. Cerebral hemodynamics can be analyzed by observing changes in oxygen saturation in the blood (Scholkmann et al., 2014). fNIRS signals are consistent with cerebral hemodynamic changes, making it a useful method to monitor cerebral perfusion during HUT (Mehagnoul-Schipper et al., 2000). Based on these basic hemodynamic principles, we sought a more comprehensive understanding of the immediate sequelae of electrical injury affecting prefrontal cerebral hemodynamics and the ANS using fNIRS. However, proper interpretation of the fNIRS signal is complex. fNIRS is influenced by hemodynamics/oxygenation occurring in the cerebral and extracerebral compartments associated with changes in HR, BP, breathing rate, CO2 concentration in the blood, and ANS activity (Tachtsidis and Scholkmann, 2016; Scholkmann et al., 2022b). Systemic physiology augmented functional near-infrared spectroscopy (SPA-fNIRS) is a method that enables the measurement and analysis of fNIRS neuroimaging data along with data from systemic physiology. SPA-fNIRS is expected to be an excellent approach to study brain-body interaction using systemic physiological signal data (Scholkmann et al., 2022a).

Several characteristics and mechanisms of an electrical burn injury can be considered from our findings. Increased sympathetic activity in the acute stage after burn injury is an important part of cardiovascular and hemodynamic compensation and wound repair. Severe burns cause long-term systemic effects, but the sequelae of non-serious burns were largely overlooked until recently (O'Halloran et al., 2016). In this study, three out of four patients with APG had total body surface area (TBSA) values less than 6, suggesting that autonomic dysfunction due to changes in sympathetic activity may occur in non-severe burns as well as severe burns. The patients included in this study were men aged 40–50 years, and most of them were affected by electrical burns due to high-voltage currents while working. TBSA varied from person to person because the resistance of each body part is a significant factor in determining the degree of damage (Christensen et al., 1980). In the human body, the resistance of the nervous system to electric current is small, and thus, in patients with electrical burns, the nervous system may be more damaged than other organs because of its good electrical conductivity (Breugem et al., 1999).

Burns can profoundly affect a patient's psychological and emotional state (Thompson and Kent, 2001). One study has shown that symptoms of anxiety and depression are related to the depth of burns (Bhatti et al., 2020). However, conflicting results were also reported that showed no difference in the incidence of anxiety or depression after burns (Nilsson et al., 2019). Our results of the questionnaires conducted before fNIRS measurements showed that patients with electrical burns experienced more severe symptoms of ANS dysfunction and depression in the acute stage compared to that of healthy participants. These results are consistent with known outcomes like psychosis, depression, and anxiety commonly observed during the acute stage of electrical burn injuries (Andrews and Reisner, 2017). In the present study, electrical burns were not associated with anxiety, and the association between depression and burn characteristics was not further analyzed. Long-term follow-up for anxiety and depression is required for patients with burns, and the occurrence of such symptoms should be reflected in treatment strategies. Future efforts are needed to explore the relationship between brain responses and psychiatric disorders in patients with burns using fNIRS. The K-COMPASS 31 scores showed differences by group, but there was no difference in OI analyzed in relation to HUT. This suggests that autonomic dysfunctions in the vasomotor, secretomotor, gastrointestinal, bladder, and pupillomotor functions other than OI exist due to electrical burns.

This study had several limitations. First, the sample size was small because this was a study on fNIRS application in patients with electrical burns. It is challenging to include a large number of patients with acute electrical injuries in a single institution due to the difficulty of performing HUT, the instability of vital signs, and the difficulty of using this fNIRS approach in patients with forehead skin burns. Although the sample size is small for each subgroup, we carefully considered selecting only patients who best fit the definition of their diagnosis. We also omitted subjects with any history of central nervous system disorder, which may affect the autoregulation function. Second, the measurements did not reflect whole-brain oxygenation because the device only detected oxygenation in the forehead area. Different optical channels were evaluated at various distances; however, only the 3 cm channel was used for comparison in this study. As it is a global change in the frontal lobe, the hemodynamic changes in the scalp can also affect the overall frontal hemodynamic changes; however, it is not expected to significantly affect the results as in other studies (Kim et al., 2018a,b, 2019). Third, most patients were not systematically followed up. We did not measure fNIRS signals after the acute period, and thus, could not compare them across other time periods. Finally, we considered the motion correction algorithm before extracting the result, but some data showed a continuously increasing tendency caused by over-compensation. This tendency contaminated the change of hemodynamic signal varied by posture changes. Thus, we did not apply the motion correction algorithm. Further studies with a larger number of patients are needed to confirm the clinical usefulness and effectivenesse of fNIRS.

## Conclusions

Electrical burns may affect cerebral perfusion, autonomic dysfunction, and depression during the acute stage of electrical injuries. The addition of fNIRS to HUT measurements better detected cerebral hemodynamic changes for patients with early electrical burns than traditional methods. Future research using fNIRS should be conducted to broaden the understanding of mechanisms, treatment, and prognosis for electrical burns.

## Data availability statement

The raw data supporting the conclusions of this article will be made available by the authors, without undue reservation.

## Ethics statement

All participants provided written informed consent before inclusion in the study. All procedures were performed in accordance with the Declaration of Helsinki and were approved by the Hallym University Institutional Review Board (IRB No. 2018-058). The patients/participants provided their written informed consent to participate in this study.

## Author contributions

YHK and B-JK designed the study. YHK, YK, JY, YC, DK, JH, and WC conducted the data collection. YHK interpreted the data and wrote this manuscript. All authors reviewed the first manuscript draft, critically revised the manuscript, read, and approved the final version.

## Funding

This work was supported by the National Research Foundation of Korea (NRF) grant funded by the Korea Government (MSIT) (No. NRF-2019R1G1A1010030). This research was also supported by a grant of the Korea Health Technology R&D Project through the Korea Health Industry Development Institute (KHIDI), funded by the Ministry of Health & Welfare, Republic of Korea (Grant Number: HI14C3477). The funders had no role in study design, data collection, analysis, decision to publish, or preparation of the paper.

## Conflict of interest

The authors declare that the research was conducted in the absence of any commercial or financial relationships that could be construed as a potential conflict of interest.

## Publisher's note

All claims expressed in this article are solely those of the authors and do not necessarily represent those of their affiliated organizations, or those of the publisher, the editors and the reviewers. Any product that may be evaluated in this article, or claim that may be made by its manufacturer, is not guaranteed or endorsed by the publisher.
