# Supplementary material for: Frontal lobe hemodynamics detected by functional near-infrared spectroscopy during head-up tilt table tests in patients with electrical burns
Source: Front Hum Neurosci. 2022 Sep 8;16:986230. doi: 10.3389/fnhum.2022.986230 (PMC9493373; doi:10.3389/fnhum.2022.986230)
Supplement: Supplementary file 1 [file Table_1.DOCX]

# Supplementary Table

The estimated MNI coordinates for each channel and Brodmann area.

| **Channel** | **X** | **Y** | **Z** | **Location** | **Brodmann Area** |
| --- | --- | --- | --- | --- | --- |
| CH 1 | 48.3 | 38.3 | 33.3 | Right | Dorsolateral Prefrontal Cortex |
| CH 2 | 40.3 | 48.3 | 32.3 | Right | Dorsolateral Prefrontal Cortex |
| CH 3 | 31.7 | 55.7 | 31.7 | Right | Dorsolateral Prefrontal Cortex |
| CH 4 | 61.0 | 22.7 | 21.3 | Right | Ventrolateral Prefrontal Cortex |
| CH 5 | 56.7 | 34.3 | 20.7 | Right | Dorsolateral Prefrontal Cortex |
| CH 6 | 49.7 | 46.3 | 20.7 | Right | Dorsolateral Prefrontal Cortex |
| CH 7 | 42.3 | 56.3 | 20.3 | Right | Frontopolar prefrontal Cortex |
| CH 8 | 33.7 | 63.0 | 19.0 | Right | Frontopolar prefrontal Cortex |
| CH 9 | 60.0 | 27.7 | 7.3 | Right | Ventrolateral Prefrontal Cortex |
| CH 10 | 57.0 | 38.3 | 6.3 | Right | Ventrolateral Prefrontal Cortex |
| CH 11 | 50.0 | 51.0 | 6.0 | Right | Dorsolateral Prefrontal Cortex |
| CH 12 | 43.7 | 60.0 | 6.0 | Right | Frontopolar prefrontal Cortex |
| CH 13 | 33.3 | 67.0 | 5.7 | Right | Frontopolar prefrontal Cortex |
| CH 14 | 50.0 | 51.3 | -8.3 | Right | Orbitofrontal Cortex |
| CH 15 | 42.7 | 60.7 | -7.7 | Right | Orbitofrontal Cortex |
| CH 16 | 33.7 | 67.0 | -7.7 | Right | Orbitofrontal Cortex |
| CH 17 | 21.0 | 62.0 | 31.7 | Right | Dorsolateral Prefrontal Cortex |
| CH 18 | 11.0 | 65.0 | 32.0 | Right | Dorsolateral Prefrontal Cortex |
| CH 19 | -2.3 | 63.0 | 31.7 | Left | Dorsolateral Prefrontal Cortex |
| CH 20 | -14.3 | 62.7 | 32.7 | Left | Dorsolateral Prefrontal Cortex |
| CH 21 | 22.0 | 69.7 | 19.0 | Right | Frontopolar prefrontal Cortex |
| CH 22 | 11.7 | 71.0 | 19.3 | Right | Frontopolar prefrontal Cortex |
| CH 23 | -2.7 | 68.7 | 17.7 | Left | Frontopolar prefrontal Cortex |
| CH 24 | -15.0 | 70.0 | 19.0 | Left | Frontopolar prefrontal Cortex |
| CH 25 | 22.3 | 72.0 | 5.7 | Right | Frontopolar prefrontal Cortex |
| CH 26 | 11.7 | 74.0 | 5.7 | Right | Frontopolar prefrontal Cortex |
| CH 27 | -3.7 | 71.0 | 5.3 | Left | Frontopolar prefrontal Cortex |
| CH 28 | -15.3 | 73.0 | 5.3 | Left | Frontopolar prefrontal Cortex |
| CH 29 | 21.7 | 71.0 | -6.7 | Right | Orbitofrontal Cortex |
| CH 30 | 11.3 | 73.0 | -7.7 | Right | Orbitofrontal Cortex |
| CH 31 | -2.3 | 70.0 | -7.7 | Left | Orbitofrontal Cortex |
| CH 32 | -14.7 | 71.0 | -8.7 | Left | Orbitofrontal Cortex |
| CH 33 | -26.0 | 56.7 | 32.0 | Left | Dorsolateral Prefrontal Cortex |
| CH 34 | -37.7 | 48.7 | 32.3 | Left | Dorsolateral Prefrontal Cortex |
| CH 35 | -46.7 | 37.7 | 32.3 | Left | Dorsolateral Prefrontal Cortex |
| CH 36 | -26.7 | 65.0 | 19.7 | Left | Frontopolar prefrontal Cortex |
| CH 37 | -38.3 | 57.7 | 19.7 | Left | Frontopolar prefrontal Cortex |
| CH 38 | -47.7 | 45.3 | 19.7 | Left | Dorsolateral Prefrontal Cortex |
| CH 39 | -55.3 | 31.7 | 18.3 | Left | Dorsolateral Prefrontal Cortex |
| CH 40 | -60.0 | 19.3 | 19.3 | Left | Ventrolateral Prefrontal Cortex |
| CH 41 | -26.7 | 68.0 | 4.3 | Left | Frontopolar prefrontal Cortex |
| CH 42 | -39.0 | 61.7 | 4.3 | Left | Frontopolar prefrontal Cortex |
| CH 43 | -48.3 | 49.7 | 4.3 | Left | Dorsolateral Prefrontal Cortex |
| CH 44 | -55.0 | 36.7 | 3.7 | Left | Ventrolateral Prefrontal Cortex |
| CH 45 | -58.0 | 23.7 | 5.3 | Left | Ventrolateral Prefrontal Cortex |
| CH 46 | -26.7 | 67.0 | -9.7 | Left | Orbitofrontal Cortex |
| CH 47 | -38.3 | 60.7 | -10.3 | Left | Orbitofrontal Cortex |
| CH 48 | -47.7 | 49.3 | -10.7 | Left | Orbitofrontal Cortex |
